# 

**Published:** 1856-08

**Authors:** 


CONTENTS.

ORIGINAL COMMUNICATIONS.
Medical Etiquette—G. W. Hall,....241

SELECTIONS,
Annual Address of Am. Med. Ass...245
Infantile Therapeutics,............256
Experiments in Cholera,.... ......265
Albumen—Translation,............369
Ammonia and Ammoniacal Salts,...270
Address of Dr. Durkee,............272
Transactions of Iowa Med. Society,.273

EDITORIAL.
Prof. Daniel Meeker,..............305
Announcements,....................305
Bibliographical Notices,...........308

Seventh Annual Announcement of the Medical Department of the Iowa University................313

